# Clinical, radiological and electrophysiological predictors for drug-resistant epilepsy

**DOI:** 10.1186/s41983-023-00647-1

**Published:** 2023-03-29

**Authors:** Noha T. Abokrysha, Noha Taha, Reham Shamloul, Samar Elsayed, Wesam Osama, Ghada Hatem

**Affiliations:** 1 Department of Neurology, Faculty of Medicine, Cairo University, Giza, Egypt; 2 Department of Internal Medicine, Faculty of Medicine, Cairo University, Giza, Egypt

**Keywords:** Drug-resistant epilepsy, Clinical predictors, MRI brain, EEG abnormalities

## Abstract

**Background:**

Epilepsy is the third chronic brain illness worldwide. About a third of the epileptic patients will be drug resistant. Early identification of these patients is critical for appropriate treatment selection and prevention of the devastating consequences of recurrent seizures. The objective of this study aims to detect clinical, electrophysiological, and radiological predictors for drug-resistant epilepsy patients.

**Results:**

One hundred fifty-five patients were included in this study, divided into a well-controlled epilepsy group (103 patients) and a drug-resistant group (52 patients). Both groups were compared regarding clinical, electrophysiological, and neuro-radiological data. Younger age at onset, history of delayed milestones, history of perinatal insult (especially hypoxia), mental retardation, neurological deficits, depression, status epilepticus (SE), complex febrile seizures, focal seizure to bilateral tonic–clonic convulsion as well as multiple seizures and high seizure frequency (daily) at onset, poor response to first anti-seizure drug (ASD), structural and metabolic etiology, abnormal brain imaging, and slow background and multifocal epileptiform discharges in EEG were significant risk factors for the development of drug-resistant epilepsy.

**Conclusion:**

MRI abnormalities are the most significant predictor for drug-resistant epilepsy. Drug-resistant epilepsy is associated with clinical, electrophysiological, and radiological risk factors that can be used to diagnose drug-resistant patients early and choose the best treatment option and time.

## Background

Epilepsy is the third chronic brain illness worldwide [1]. About a third of the epileptic patients will be drug resistant [2], and 80% of them are in developing countries [3]. Drug-resistant patients have a higher risk of developing psychosocial, psychiatric, and medical morbidities, which must be addressed promptly to improve their quality of life [4]. Early identification of drug-resistant epilepsy is essential for proper treatment selection to prevent the overwhelming effects of drug-resistant epilepsy [5].

Particular definition of drug-resistant epilepsy has remained vague, resulting in different criteria used by different clinicians and researchers so, the International League Against Epilepsy (ILAE) proposed a definition to improve patient care and enable clinical research. ILAE defines drug-resistant epilepsy as “failure of adequate trial of two tolerated, appropriately chosen and used antiepileptic drug (whether as monotherapies or in combination) to achieve sustained seizure freedom for more than 1 year, or having sporadic seizures separated by a period three times the longest interval between seizures prior to the treatment, either is longer [2].

Among the different suggested definitions of drug-resistant epilepsy before that of ILEA that had a good inter- and intra-observer agreement was that of Camfield and colleagues that defined patients who, over the previous year of observation, had an average of two or more seizures every 2-month interval, while receiving at least 3 anti-seizure drugs (ASD) as monotherapy or polytherapy as drug resistant [6].

Numerous studies have investigated the factors that predict drug resistance in children and adults. The following are the risk factors most often linked to drug-resistant epilepsy: mental retardation, neurological abnormalities, younger onset age, symptomatic etiology, high-frequency seizures, non-response to the initial ASD, and abnormal electroencephalography (EEG) findings [2]. This study aims to detect clinical, electrophysiological, and radiological findings that could be used as early predictors for drug-resistant epilepsy.

## Methods

The current retrospective longitudinal case–control study was conducted at a tertiary referral center. One hundred fifty-five epileptic patients were selected from the epilepsy clinic, have been following up for 2 years before recruitment and split into two groups; Drug-resistant group (52 patients, cases): patients had at least one seizure per month over 1 year despite receiving maximum tolerated dosages of two or more ASDs, and well-controlled epilepsy group (103 patients, controls): patients had one seizure or less per year with one or two ASDs.

Informed written consent to participate in the study was obtained from participants (or their parent or legal guardian in the case of children under 16).

The inclusion criteria include the following: patients above 14 years of both sexes, diagnosed as epileptic according to the 2017 ILAE definition of epilepsy. Taking one or more appropriate ASD for at least 1 year and compliant with treatment. Excluded from the study: patients with poor history reporting and cases newly diagnosed with epilepsy (< 6 months).

According to the procedure employed at the epilepsy unit, patients and their families were questioned, and pertinent information about the patient’s ailment was documented. The following procedures were applied to all patients:

Clinical assessment including: personal history: age, sex, first unprovoked seizure onset age, family history of seizure disorders and parental consanguinity, perinatal history, neurodevelopmental history (delayed milestones), initial seizure type, febrile convulsion, and status epilepticus (SE) history and seizure frequency: initial frequency, after the first ASD, and current frequency.

General and neurological examination to detect neurological deficits. An IQ test to detect mental retardation (IQ of less than 70). Depression was diagnosed based on the DSM-5. Laboratory tests were done according to medical requirements. Magnetic resonance imaging of the brain (1.5T IGC F2000 Magnet 2016 USA Philips Intera^®^ scanner) including axial T1WI, T2WI, sagittal T1WI, T2WI, FLAIR, and coronal T2WI. Electroencephalography (EEG) records using a multichannel basic EEG device. The electroencephalograph system used in our unit was EB Neuro Galileo NT machine (EBNeuro, Firenze, Italy, serial number DAUNL7HQ4NUSFG; model Mizar B8351037899; version 3.61).

Statistical comparisons were used to identify early predictors of drug resistance. All collected data were revised for completeness and accuracy. Pre-coded data were entered on the computer using the statistical package of social science software program, version 21 (SPSS) to be statistically analyzed. Data were summarized using—mean and SD for quantitative variables—number and percent for qualitative variable.

Comparison between qualitative variables was done using Chi-square test for qualitative variables while independent *T* test for quantitative variable which where normally distributed and nonparametric Kruskal–Wallis and Mann–Whitney tests for quantitative variables which were not normally distributed. Pearson correlation test was used to find univariate association between variables Multivariate analysis was used to find significant predictors for primary outcome *p* value less than 0.05 was considered of statistically significant pre-coded data were entered on the computer using the statistical package of social science software program, version 15 (SPSS) to be statistically analyzed.

## Results

The clinical predictors are summarized in Table 1.

**Table 1:** Clinical predictors for drug-resistant epilepsy

Clinical predictors	Control *n* = 103 (%)	Case n = 52 (%)	*P*-value
Sex			
Male	42 (40.8)	33 (63.5)	0.08
Female	61 (59.2)	19 (36.5)	
Age			
Mean ± SD	31.7 ± 11.5	22.2 ± 8	< 0.001*
Age at onset			
Infant (0–2)	7 (6.8)	18 (34.6)	< 0.001*
Child (2–12)	30 (29.1)	29 (55.8)	
Adolescence (2–18)	43 (41.8)	5 (9.6)	
Adult (18–65)	21 (20.4)	0	
Elderly > 65	2 (1.9)	0	
Family history of epilepsy			
Positive	38 (36.9)	26 (50)	0.118
Negative	65 (63.1)	26 (50)	
Neonatal history			
Perinatal history			
Normal	99 (96.1)	35 (67.3)	< 0.001*
Hypoxia	4 (3.9)	17 (32.7)	
Developmental milestone			
Normal	99 (96.1)	35 (67.3)	< 0.001*
Delayed	4 (3.9)	17 (32.7)	
Febrile seizures			
None	91 (88.3)	18 (34.6)	< 0.001*
Simple	11 (10.7)	5 (9.6)	
Complex	1 (1)	29 (55.8)	
Seizure type			
Myoclonus	23 (22.3)	0	< 0.001*
Focal aware	26 (25.2)	0	< 0.001*
Focal evolving to bilateral	13 (12.6)	31 (59.6)	< 0.001*
Multiple seizure types	0	21 (40.4)	< 0.001*
Tonic	2 (1.9)	0	0.551
Clonic	3 (2.9)	0	0.327
Tonic–clonic	3 (2.9)	0	0.327
Absence	3 (2.9)	0	0.327
Initial seizure frequency			
Less than 1 per year	5 (4.8)	0	0.169
Monthly	68 (66)	7 (13.5)	< 0.001*
Weekly	28 (27.2)	20 (38.5)	0.193
Daily to frequent per day	2 (1.9)	25 (48)	< 0.001*
In conclusion: patients who had more than 1 per month	29.1%	86.5%	< 0.001*
Response to the first ASD			
Less than 1 per year	82 (79.6)	0 (0)	0.003^*^
1/6 months	21 (20.4)	1 (1.9)	< 0.001*
Monthly	0 (0)	16 (30.8)	< 0.001*
Weekly	0 (0)	22 (42.3)	< 0.001*
Daily to frequent per day	0 (0)	13 (25)	< 0.001*
In conclusion: seizure reduction to 1/ year	79.6%	1.9%	< 0.001*
History of SE			
None	98 (95.1)	16 (30.8)	< 0.001*
Once	5 (4.9)	14 (26.9)	
More than 2	0 (0)	22 (42.3)	
Etiology			
Idiopathic	50.5	3.8	≤ 0.001*
Structural	16.5	38.5	0.002*
Infection	1.9	13.5	0.007*
Metabolic	0	21.2	< 0.001*
Associated comorbidity			
Neurological deficits	9 (8.7)	30 (57.7)	< 0.001*
Intellectual disability	6 (5.8)	38 (73.1)	
Depression	7 (6.8)	19 (36.5)	

*SD* standard deviation, *ASD* anti-seizure drug, *SE* status epilepticus

* statistically significant

Electrophysiological predictors revealed that significantly more abnormal EEG results were seen in the drug-resistant group (69.2%) than in the well-controlled group (44.7%). Background (BG) activity showed that 61.5% of the drug-resistant group had diffuse slowing and asynchronous BG, compared to 1.9% of the well-controlled group (*p* ≤ 0.001).

Regarding epileptiform discharges, multifocal discharges were significantly more prevalent (*p* ≤ 0.001) in the drug-resistant group (25%) than in the well-controlled group (1%). The prevalence of extratemporal epileptiform discharges was substantially greater (*p* < 0.001) in the drug-resistant group (21.2%) than in the well-controlled group (1%). Comparatively, there was no significant difference between the prevalence of temporal discharges in the drug-resistant group (17.5%) and the well-controlled group (34.6%).

Radiological predictors showed that in the drug-resistant group, abnormal brain imaging was significantly greater than in the control group (88.5% and 27.2%, respectively), indicating refractoriness. In contrast, pharmacological responsiveness is shown by the presence of a normal MRI brain in 72.8% of the well-controlled group compared to 11.5% of the drug-resistant group (*p* ≤ 0.001).

Brain atrophy was the most prevalent radiological characteristic in the drug-resistant group (especially cerebellar atrophy) in 25% and temporal sclerosis in 11.5%, with a statistically significant difference (P ≤ 0.001). Other brain findings, including vascular, demyelinating, cortical dysplasia, and space-occupying lesions, were not statistically significant between both groups.

Multiple stepwise regression found that the significant predictors for the outcome (well-controlled/drug-resistant epilepsy) included neurological deficit, mental retardation, and abnormal MRI; the most significant predictor was abnormal MRI.

## Discussion

As one-third of patients with epilepsy will not be properly controlled with ASD [2], early diagnosis of individuals at high risk of developing drug-resistant epilepsy is vital for the right selection of an effective alternative treatment to avoid the detrimental effects of recurring seizures on behavior and intellectual development and to decrease the side effects of ASD overdose and medication interactions arising from polytherapy [5]

Early-onset seizures may promote epileptogenesis in the developing brain, leading to drug resistance [7]. In the present study, 90.4% of drug-resistant patients had an onset of seizures at less than 12 years. This result agrees with previous study that found that most drug-resistant patients had the onset of seizures before 14 years [8]. Besides, the history of delayed milestones and perinatal insult was a predictor of drug resistance [9]. The association between developmental delay and negative outcomes may result from minor but widespread brain anatomical abnormalities [10]. Hypoxic or asphyxic events, mainly in the perinatal period, promote epileptogenesis and are important in developing drug resistance [11]

In the present study, a positive family history of seizures was not identified as a predictor of drug resistance. This is consistent with Ayca and colleagues [11], who showed that a positive family history of seizures did not seem to be a risk factor for drug-resistant epilepsy. Other studies found no significant correlation between a family history of seizures and drug-resistant epilepsy [10, 12, 13]. Twin studies suggested that genes and, therefore, family history may have a role in the pathophysiology of epilepsy but not in its prognosis [14].

In the present study, we found that neurological deficits and depression were higher in the drug-resistant group. These outcomes are similar to previous studies [9, 15]. In addition, previous studies also showed that neurological impairment could alone predict drug resistance [16]. Ayca and colleagues [11] also found that abnormal neurological examination was 58 times more in drug-resistant epilepsy and was the most important risk factor, possibly reflecting the severity of brain damage. The presence of abnormal neurological examination agrees with other studies [10, 17]. Drug-resistant epilepsy has been linked to a history of psychiatric comorbidity, particularly depression, according to Hitiris and colleagues [14]. It is hypothesized that both share a pathophysiological mechanism that increases the severity of brain malfunction and the risk of developing drug-resistant epilepsy.

Furthermore, we found that intellectual disability was higher in the drug-resistant group, as found in previous studies [9, 15]. Intellectual disability was significantly more observed in the drug-resistant group and was found as an independent indicator for predicting drug resistance [13].

High seizure frequency occurring immediately after the diagnosis of epilepsy, either before or after the initiation of therapy, is associated with short- and long-term drug resistance [18].

In the current study, high seizure frequency (daily to monthly) occurring either before or after treatment was higher in the drug-resistant group. This result agrees with several studies that showed a strong positive association between high initial seizure frequency and drug resistance [9, 15, 19]. Frequent seizures result in mossy fiber sprouting and neurodegeneration in the hippocampus, producing recurrent excitatory circuits [20]. Moreover, the “intrinsic severity hypothesis” assumes that pharmacoresistance is related to epilepsy severity, which is reflected in seizure frequency before the onset of ASD therapy and associated with a poor likelihood of long-term seizure remission after therapy [21].

Although it is unclear if ASD failure relates to the underlying cause of epilepsy or a patient-specific genetic trait, responsiveness to the first administered ASD is a prognostic factor and a predictor of drug resistance [22]. The outcomes of previous studies have shown that after an unsuccessful first ASD, the chance of seizure control is lowered by 4–5 times and becomes low (1–3%) after an ineffective second ASD [23, 24].

In the current study, high seizure frequency (daily or monthly) after receiving the first ASD was higher in the drug-resistant group. This comes in accordance with Tripathi and colleagues [8], who found that response failure to the first ASD was the second prognostic factor in developing drug-resistant epilepsy. Dlugos and colleagues [25] stated that failure of the first ASD predicts drug resistance in temporal lobe epilepsy (TLE) in children.

The “network hypothesis” proposes that seizure-induced structural brain alterations, such as synaptic reorganization, axonal sprouting, gliosis, and neurogenesis, can contribute to forming an abnormal neural network that prevents ASD from entering its targets, thereby resulting in drug-resistant epilepsy. Moreover, the “gene variant hypothesis” proposes an innate resistance governed by genetic variations of proteins implicated in the pharmacokinetics and pharmacodynamics of ASD activities [26, 27].

We observed that the type of seizure was a predictor of seizure outcome. This finding was reported in previous studies [8, 15], additionally, it has been hypothesized that focal epilepsy is a strong predictor of the prevalence of drug-resistant epilepsy [28]. Focal seizure to bilateral tonic–clonic seizure and multiple seizure types predicts drug resistance. Besides, the generalized tonic–clonic seizure was predictive of treatment response, and this considerable link between focal epilepsy and drug resistance may represent structural/metabolic factors within the drug-resistant group [15]. Flores-Sobrecueva and colleagues [29] stated that the possibility to develop drug-resistant epilepsy is higher in patients with focal onset seizures compared to the generalized onset, with focal onset seizures representing 94% while generalized onset represented 6%.

Multiple seizure types were identified as significant risk factors for drug resistance [20]. Yilmaz and colleagues [30] reported that Patients with drug-resistant epilepsy are more likely to experience mixed seizures than drug-responsive patients, who are more likely to experience generalized seizures. Similarly, a retrospective study showed that mixed seizure is an independent risk factor for drug-resistant epilepsy in childhood [31]. Chawla and colleagues [32] discovered that generalized tonic seizure was the most prevalent type in the drug-resistant epilepsy group.

SE represented a predictive factor for drug resistance. This came in accordance with previous studies [8, 15, 16, 31]. In contrast, Saygi and colleagues [9] reported that SE had insignificant predictive value. SE was caused by reduced inhibition and hyper-excitability; as SE persisted longer, gamma-aminobutyric acid function diminished and excitatory input continued, contributing to neurodegeneration, boosting epileptogenesis, and increasing drug resistance [33].

The association between drug-resistant epilepsy and febrile seizures is controversial. In our study, febrile seizures (especially complex seizures) were higher in the drug-resistant group. Similar findings were made by Tripathi and colleagues [8], who noted that drug-resistant epilepsy was related to extended febrile seizures. This can be due to the association of febrile seizures with severe damage to the temporomesial structures, leading to brain dysfunction [20], and that hyperthermia can cause hippocampal damage [13]. In contrast, Saygi and colleagues [9] found no relation between febrile seizures and seizure outcomes.

Although the EEG is commonly conducted during the early stages of diagnosis, few studies have correlated the EEG results to the outcome. According to several studies, abnormal EEG activity is associated with a worse prognosis. In addition, drug resistance was shown to be predicted by abnormal EEG (slow wave and epileptiform discharges) [20]. In our study, abnormal EEG findings were more prevalent among drug-resistant patients and refractoriness is predicted by background activity (diffuse slowness or asynchronous background) and epileptiform discharge (particularly multifocal discharge). Besides it was found that strong predictors of drug-resistant epilepsy were multifocal epileptiform discharges [34].

Comparing etiological causes between drug-resistant and well-controlled groups, we observed that idiopathic causes were a good indicator for drug response, while symptomatic etiology strongly predicted drug resistance. Symptomatic etiology has been described as an important risk factor for drug-resistant epilepsy [9, 30]. In this study, structural etiology, metabolic causes, and infections were the commonest etiological causes in the drug-resistant group. Similarly, Farghaly and colleagues [15] reported that structural/metabolic etiology was strongly associated with drug resistance. Also, Shan and colleagues [35] reported that the most frequent cause of drug-resistant epilepsy was structural.

This was supported by MRI findings, where abnormal brain imaging was the most important predictor of drug-resistant epilepsy in this study. In contrast, a normal MRI brain was more in a well-controlled group, suggesting a good indicator for drug responsiveness. Previous studies have revealed that abnormal MRI findings predict drug resistance [30]. In addition, children with drug-resistant epilepsy had significantly more MRI abnormalities [31].

Brain atrophy was the most prevalent radiological characteristic in the drug-resistant group (especially the cerebellum) and temporal sclerosis. According to the transporter theory, structural defects injure the capillary endothelial cells that comprise the blood–brain barrier, resulting in an overexpression of efflux transporters and drug resistance [20].

Our results were close to the study performed by Tripathi and colleagues [8], who stated that the most common radiological features were perinatal hypoxic–ischemic brain injuries, dysembryoplastic neuroepithelial tumors, and mesial temporal sclerosis. Liu and colleagues [36] reported significant atrophy of the neocortex, hippocampus, or cerebellum in 17% of drug-resistant patients. Cerebellar atrophy causes loss of inhibitory function in the brain and worsens the prognosis of epilepsy. It could result from seizure-mediated cellular damage, prolonged drug therapy (especially phenytoin), SE, or hypoxic damage [36].

## Conclusion

In this study, several clinical, electrophysiological, and radiological factors can help to predict patients at risk of developing drug-resistant epilepsy so that close monitoring and early introduction of alternative treatment options can help to prevent the consequences of frequent seizures or ASD side effects and interactions. The most important were abnormal MRI and the presence of neurological deficits on examination.

The limitation of this study is sample size was relatively small. It was done in a tertiary hospital during the COVID-19 pandemic, influencing the frequency of seizures and the number of recruited patients. Being a case–control study, it could not differentiate between the causes and consequences of some predictors. Moreover, limited resources for better assessing refractory patients regarding brain imaging and EEG.

## Data Availability

All data generated or analyzed during this study are available from the corresponding author on reasonable request.

## Abbreviations

ASD: Anti-seizure drug
BG: Background
DNET: Dysembryoplastic neuroepithelial tumor
EEG: Electroencephalography
GABA: Gamma aminobutyric acid
ILAE: International League Against Epilepsy
MTS: Mesial temporal sclerosis
SE: Status epilepticus
TLE: Temporal lobe epilepsy
